# Supplementary material for: Cross-Sectional Survey of the Training Practices of Racing Greyhounds in New Zealand
Source: Animals (Basel). 2020 Nov 4;10(11):2032. doi: 10.3390/ani10112032 (PMC7694201; doi:10.3390/ani10112032)
Supplement: Supplementary file 1 [file animals-10-02032-s001.pdf]

MASSEY UNIVERSITY
TE KUNENGA KI PŪREHUROA
UNIVERSITY OF NEW ZEALAND

SCHOOL OF VETERINARY SCIENCE

Training survey of New Zealand racing greyhounds

Massey University Doctoral student, Anna Palmer, and supervisors Dr Charlotte Bolwell, Associate Professor Chris Roger, Professor Kevin Stafford and Dr Arnon Gal are conducting a survey to understand the training of Greyhounds in New Zealand.

- This survey contains questions about the greyhounds you train. Answers are generalised so please consider a **typical greyhound** when completing the questions.

- Questions require you to either tick the boxes, or provide details in the form of a written answer.

- Please consider all options before indicating the most appropriate response to each and every question.

- This survey should only take **15 minutes** to complete.

- All responses are completely **confidential**. Data will be used for research purposes only.

- By completing this survey, you are giving your consent for the information you give to be used as part of this research.

- Please return the survey by post as soon as possible and before **4th October** 2019.

Thank you for your assistance!

Greyhound training survey

How many dogs are you currently training TODAY? ☐☐☐

Which type of training license do you hold?

- Public ☐
- Owner/Trainer ☐

What is your age?

- ≤20 years ☐
- 21-30 years ☐
- 31-40 years ☐
- 41-50 years ☐
- 51-60 years ☐
- 61-70 years ☐
- 71-80 years ☐
- 81≥ years ☐

What is your gender?

- Male ☐
- Female ☐

How many years have you been training greyhounds? ☐☐

What facilities do you use to train your dogs? Please fill out the following table by circling the appropriate answer or providing details where applicable.

**Run or slipping track**	Y / N	Distance of run:	Straight / Curved	Flat / Hill
**Exercise paddock**	Y / N	Size (h):		
**Circular training track**	Y / N	Diameter:		
**Bull Ring**	Y / N	Diameter:		
**Local race track**	Y / N	Track name:		
**Beach**	Y / N			
**Treadmill**	Y / N			
**Starting box(es)**	Y / N			
**Other facilities** (Please specify)				

Section 1 – Training before dogs begin racing

If you prepare young dogs for racing or train dogs before they begin racing, please answer the questions BELOW.

If you have only trained dogs that are in race work, please go to Section 2 (page 6).

What is the primary reason for deciding when to register a dog for racing? (Select one)

- Overall appearance of the dog (looks fit and healthy) ☐
- Capable of meeting time milestones ☐
- Been in training for an appropriate number of weeks (please specify) ☐ ____weeks
- Owner decision ☐
- All dogs in training are registered for racing ☐
- Age of the dog ☐
- Other (please specify) ____

Do your dogs follow a standard training programme up to their first race?

- Standard training programme for all dogs ☐
- Similar training programme with minor changes for each dog ☐
- Different training programme for each dog ☐

At what age (months) do dogs typically begin training? ☐☐ months

Typically, <u>from entering training</u>, how many weeks does it take for a dog to reach the following milestones? (Please state n/a if not applicable to your programme)

**Milestone**	**Number of weeks from entering training**
Learning to chase	
Fast work	
Box training	
Hand slip on track or partial trials	
Full trials before a break	
Full trials after a break	
Qualifying trial	
First Race	

Do your young dogs have a break during the breaking in process?

No ☐

Yes – please state the length of break: ______________________________________

When or at what stage does this occur? ______________________________________

Do you utilise trials (not qualifying trials) as part of a dog's training programme?

No ☐

Yes – Primary reason:

- to educate the dog ☐
- to improve fitness ☐
- for another reason: please state: ______________________________________

How many full trials would a dog typically have before its qualifying trial? ☐☐

What is the primary reason for deciding when a dog is ready for a qualifying trial? (Select one)

- Overall appearance of the dog (looks fit and healthy) ☐
- Capable of meeting time milestones ☐
- Been in training for an appropriate number of weeks (please specify) ☐ ____weeks
- Owner decision ☐
- All dogs in training complete a qualifying trial ☐
- Age of the dog ☐

Other (please specify) ____

Section 2 – Training that occurs while the dogs are racing

The following question relates to the typical weekly training programme for a dog. The table below provides an example of how to complete this question.

	Monday	Tuesday	Wednesday	Thursday	Friday	Saturday	Sunday
**Type of training**<br>For example:<br>Gallop<br>Walk<br>Race<br>Play<br>Free exercise<br>Other (Please specify)	Racing		Walk<br><br>Free exercise	Gallop	Free exercise		
**Racing** (please tick)	✓						
**Day off** (please tick)		✓				✓	✓
**Location**<br>For example:<br>Race Track<br>Run<br>Paddock<br>Beach<br>Bull Ring<br>Other (Please specify)	Race Track		Walk = Treadmill<br><br>Free exercise = exercise paddock	Run	Paddock		
**Distance**	457m		Walk = 2km<br><br>Free ex. = N/A	200m	N/A		
**Frequency**<br>i.e. number of times activity performed	1		Walk = 1<br><br>Free ex. = 1 morning & 1 night	1	2 (1 Morning and 1 Night)		
**Duration**	20 seconds		Walk = 20mins<br><br>Free ex. = 5 mins each	15 seconds	20 minutes each		
**Other comments**							

EXAMPLE

Using the following table, please outline the typical weekly training programme for a dog, in full racing fitness, based around race-day(s). Please see the previous table for an example:

	Monday	Tuesday	Wednesday	Thursday	Friday	Saturday	Sunday
**Type of training**<br>For example:<br>Gallop<br>Walk<br>Race<br>Play<br>Free exercise<br>Other (Please specify)							
**Racing** (please tick)							
**Day off** (please tick)							
**Location**<br>For example:<br>Race Track<br>Run<br>Paddock<br>Beach<br>Bull Ring<br>Other (Please specify)							
**Distance**							
**Frequency**<br>i.e. number of times activity performed							
**Duration**							
**Other comments**							

Does your weekly training programme differ for sprinting dogs, middle distance dogs or staying dogs?

Yes No

If yes, please briefly describe the changes you make to the training programme and why you make these changes?

Do you make changes to a dog's training programme in the 48 hours before a race?

Yes No

If yes, please briefly describe the changes you make and explain why you make these changes?

What method do you use to record training sessions?

- I do not record training sessions ☐
- I record training sessions on paper or in a diary ☐
- I record training sessions in an electronic format or on a spreadsheet ☐

Other (please specify) ____

If you record training sessions, which of the following do you record?

- Time ☐
- Type of work ☐
- Frequency of work ☐
- Distance ☐

Other (please specify) ____

Thank you

…for taking the time to participate in this survey. We appreciate your time and effort!

Please **post** the survey to Anna Palmer in the **pre-paid envelope** attached.

School of Veterinary Science
Private Bag 11222, Palmerston North 4442, New Zealand **T** 06 350 4525 **F** 06 355 7957 **www.massey.ac.nz**
